# Ecotoxicological Effect of *Kappaphycus alvarezii* Extract on *Zea mays* and *Ceriodaphnia silvestrii*

**DOI:** 10.1007/s00128-026-04229-8

**Published:** 2026-03-28

**Authors:** Lucas Adriano Moreira, Erika dos Santos Silva, Antonio Rodrigues da Cunha Neto, Anelise Vieira Rosa Fernandes da Silva, Alexandra dos Santos Ambrósio, João Vitor Barbosa Calvelli, Gabriela Ezequiel Costa Martins, Maria José dos Santos-Wisniewski, Breno Régis Santos, Sandro Barbosa

**Affiliations:** Instituto de Ciências da Natureza, Universidade Federal de Alfenas, Alfenas, Minas Gerais Brazil

**Keywords:** Biostimulants, Bioassays, Maize, Cladocera

## Abstract

Marine algae are recognized as promising sources of natural biostimulants in agriculture, with potential applications in seed physiological conditioning. However, the environmental implications of their production, management, and disposal remain insufficiently addressed. This study evaluated the ecotoxicological effects of a commercial *Kappaphycus alvarezii* extract on *Zea mays* and *Ceriodaphnia silvestrii*. Conditioning assays with maize seeds were performed at extract concentrations of 0, 25, 50, 75, and 100%, assessing germination, vigor, and early seedling growth. Acute toxicity tests with *C. silvestrii* exposed individuals to 0.1–4% concentrations, and immobility was recorded to determine the median effective concentration (EC_50_). Concentrations up to 50% did not impair germination, with 25% promoting significant vegetative growth, while higher concentrations reduced germination due to phytotoxicity. In *C. silvestrii*, 2.06% caused 50% mortality. These results demonstrate the dual role of algal extracts, highlighting agronomic potential alongside ecotoxicological risks, and reinforce the need for safe production, management, and environmentally responsible disposal practices to ensure sustainability.

**Supplementary Information:**

The online version contains supplementary material available at 10.1007/s00128-026-04229-8.

## Introduction

Seed physiological priming has been widely studied as a strategy to improve germination, early vigor, and tolerance to environmental stresses. This process involves exposing seeds or seedlings to different biochemical or physical agents that stimulate their physiological and biochemical responses, resulting in more efficient development (Bewley et al. 2013).

This technique occurs in two main phases. Phase 1 involves seed imbibition, characterized by rapid water uptake, the activation of cellular metabolism, and the resumption of respiration, preparing the seed for germination. Phase 2 is marked by the stabilization of water uptake and the onset of metabolic reorganization, including enzyme activation (e.g., increased antioxidant enzyme activity) and the mobilization of energy reserves. This overall process allows for faster and more uniform germination upon subsequent sowing, which is usually observed as quicker radicle protrusion (Bewley et al. 2013; Xiong et al. 2021). Among the priming agents used, biostimulants have gained prominence due to their ability to enhance reserve mobilization, DNA repair, and serve as more accessible macromolecule precursors for seeds (Hussain et al. 2016).

Biostimulants derived from marine algae extracts, such as those from *Kappaphycus alvarezii* (Doty) Doty ex P.C. Silva, are widely recognized for their plant growth-promoting potential and ability to enhance resistance to adverse conditions. These products contain a variety of bioactive compounds, including phytohormones, polysaccharides, amino acids, and antioxidants, which play essential roles in regulating plant metabolism. The application of biostimulants can result in increased germination rates, enhanced root and leaf development, and improved resistance to both biotic and abiotic stresses (Cocetta et al. 2022). However, the effect and mode of action of biostimulants may vary depending on their bioactive composition, concentration, and application timing. Moreover, the efficacy of a biostimulant product may differ according to plant species and environmental growth conditions (Franzoni et al. 2023).

Phytotoxicity caused by improper use of biostimulants can impair plant development and result in deleterious effects such as leaf necrosis, growth reduction, and physiological imbalances. This primarily occurs when high doses are applied, which may trigger undesirable plant responses. Furthermore, compounds present in biostimulants can interact negatively with other agricultural inputs, exacerbating adverse effects on biota through leaching into fluvial ecosystems (Franzoni et al. 2023). Thus, determining safe and effective dosages is crucial to ensure the benefits of these products without compromising productivity and plant health. Moreover, the intensive application of these products in agriculture, particularly if mismanaged, can lead to off-target effects, posing a risk to adjacent ecosystems.

Therefore, the environmental impact of waste resulting from the disposal of *K. alvarezii* extracts after physiological priming may occur through the release of bioactive substances into soil and water bodies, affecting microbial communities, aquatic organisms, and even trophic chains (Vaghela et al. 2022). Thus, the application of biostimulants must be accompanied by strategies to minimize environmental impacts. Despite the growing use of marine biostimulants based on seaweed extracts, primarily red and brown algae, few studies have evaluated their post-application ecotoxicological effects on aquatic systems. Among the most widely used species, in addition to *Kappaphycus alvarezii*, the species *Ascophyllum nodosum*, *Laminaria*, *Ecklonia maxima*, and *Sargassum* stand out (Gandhi et al. 2024).

A notable bioactive compound present in these biostimulants is carrageenan, a polysaccharide found in high concentrations in *K. alvarezii* that has raised concerns regarding its toxicity (Prajapati et al. 2014). Studies have demonstrated that this component can harm aquatic organisms when used in large quantities over prolonged periods (Bhattacharyya et al. 2015; Yuan et al. 2020; Andrade et al. 2022). The toxic effects of carrageenan stem from its high molecular weight, which can induce toxicity by interfering with cellular functions and ionic balance in organisms. For microcrustaceans, the physical properties of carrageenans, specifically their high viscosity and negative charge, may interfere with osmotic balance, ultimately causing mortality (Damayanti et al. 2025).

Although they are considered natural products with relatively lower environmental impacts, it is crucial that commercial formulations be tested for their ecotoxicological potential. Therefore, bioassays are essential tools for assessing potential adverse environmental effects. These assays enable the identification of acute chemical toxicity in various organisms, including plants, microcrustaceans, fish, algae, and bacteria, providing critical data for regulatory decisions regarding compound use (Silva et al. 2020). Furthermore, bioassays contribute to understanding toxicity mechanisms and establishing safe application thresholds for agricultural biostimulants (Zhou et al. 2019).

Consequently, ecotoxicological studies are essential to ensure the sustainable use of biostimulants and the preservation of natural ecosystems. In this context, among the widely used models for toxicity assessments are *Zea mays* (Yang et al. 2015) for higher plants and *Ceriodaphnia silvestrii* for evaluating aquatic ecosystem ecotoxicity (Leite et al. 2022). Thus, since seaweed extract is already a commercial product used in the field, the guiding hypothesis is that at low concentrations (matching commercial recommendations) it would act as a biostimulant, enhancing initial growth, whereas high concentrations would be phytotoxic and inhibit *Zea mays* germination. Regarding *C. silvestrii*, the extract may exhibit toxicity, confirming the potential ecotoxicological risk of its disposal in aquatic ecosystems due to its high salinity.

Given this background, the present study aimed to: (1) perform physiological priming of *Zea mays* seeds, and (2) test the toxicity of a commercial *Kappaphycus alvarezii* extract used in this agronomic technique on both (a) germination and early growth of the plant bioassay, and (b) mobility and survival of *Ceriodaphnia silvestrii*.

## Materials and Methods

### Phytotoxicity Test Using Physiological Conditioning

The physiological conditioning technique was employed to determine acute toxicity in *Zea mays* (maize) seeds on their germination and early growth. Hybrid maize seeds (NS90) were commercially obtained, and the physiological conditioning bioassay was conducted at the Laboratorio de Biotecnologia Ambiental e Genotoxicidade (BIOGEN). The maize seeds were subjected to different treatments with a commercial *Kappaphycus alvarezii* extract, which, according to the manufacturer, is a 100% organic extract rich in natural phytohormones and amino acids (Supplemental Table S1), diluted in 500 mL of water at concentrations of 0 (hydropriming), 25, 50, 75, and 100%, along with a control group of unconditioned seeds.

The concentrations used were dilutions of the commercial product (100% = undiluted extract; 25% = 1:3 extract: water dilution). This gradient was designed to establish a clear dose-response curve. This approach allows for identifying optimal biostimulant concentrations for conditioning while also determining the extract’s upper phytotoxic threshold.

For physiological conditioning, the pH and electrical conductivity of treatments were measured (Supplemental Table S2), and an orbital shaker was used for solution aeration over 16 h at 25 ± 1 °C, following the methodology described by Brar et al. (2020).

After the treatment, the seeds were washed with distilled water and dried in a forced-air circulation oven at 25 °C for 24 h. Samples of 15 seeds were collected at two time points: after conditioning and after drying. Fresh weight was then recorded, and the seeds were oven-dried at 105 °C for moisture content determination, following the standards established by Brasil (2009).

Germination was conducted on Germitest paper moistened with 2.5 times its weight in distilled water, according to Brasil (2009). Four replicates of 50 seeds each were maintained in a B.O.D.-type germination chamber at 25 °C with a 12-h photoperiod. The first germination count was performed on the fourth day, and the final count on the tenth day, following the criteria established by Brasil (2009). Germination speed (S) was determined every 24 h according to Equation (S) proposed by Chiapusio (1997). On the tenth day, all the seedlings were photographed, and root and shoot lengths were analyzed using ImageJ software.

The experiment followed a completely randomized design (CRD). Prior to statistical analysis, all data were checked for the assumption of normality of residuals using the Shapiro-Wilk test. Once the assumptions were met, the data were subjected to analysis of variance (ANOVA), and the means were compared using the Scott-Knott test at 5% significance, employing Sisvar 5.6 software (Ferreira 2019).

### Acute Toxicity Bioassay Using Microcrustaceans

The organisms used in the acute toxicity test, the microcrustacean *Ceriodaphnia silvestrii* Daday, 1902 (Crustacea, Cladocera, Daphnidae), were obtained from the Department of Evolutionary Hydrobiology at the Federal University of São Carlos, São Paulo, and subsequently acclimated for testing at the Limnology Laboratory of the Federal University of Alfenas, Minas Gerais. Cultures were maintained under controlled temperature and photoperiod (25 ± 2 °C and 12:12-h light/dark cycle) in reconstituted water with pH 7.0–7.6, conductivity of 160 µS cm^− 1^, and hardness of 48 mg L^− 1^ (as CaCO_3_). The organisms were fed daily with the algae Raphidocelis subcapitata (10^5^ cells mL^− 1^), a suspension containing yeast (0.5%) and fermented fish food (0.5%), with 1 mL L^− 1^ provided. Acute toxicity tests with the reference substance sodium chloride (NaCl) were performed monthly to assess the physiological condition of the organisms and validate the bioassay (ABNT, 2022).

The acute toxicity assays followed protocols issued by ABNT (2022). Based on preliminary test results, the concentration range for the algal extract was established as: 4, 3, 2, 1, 0.6, 0.2, and 0.1%, for which pH and electrical conductivity were also measured (Supplemental Table S3). For each concentration, five replicates and a control (reconstituted water) were used. The experiment was conducted in non-toxic polypropylene cups containing five neonates (6–24 h old) in 20 mL of test solution or reconstituted water (control), maintained in a B.O.D.-type growth chamber at 25 ± 2 °C, without food addition and in complete darkness. After 48 h of exposure, organisms were examined under a stereomicroscope, and the number of immobile individuals was counted and used to determine the median effective concentration (48 h EC_50_). Water quality parameters (pH, temperature, electrical conductivity, and dissolved oxygen) were measured at both the beginning and end of the toxicity tests conducted according to ABNT (2022) protocols.

For data analysis, 48 h EC_50_ values were calculated through nonlinear regression using a three-parameter logistic curve in SigmaPlot 12.3 software. Normality (Shapiro-Wilk test) and variance (t-test) tests were performed. For all statistical tests, differences were considered significant when *p* ≤ 0.05.

## Results

### Phytotoxicity

**Fig. 1:**
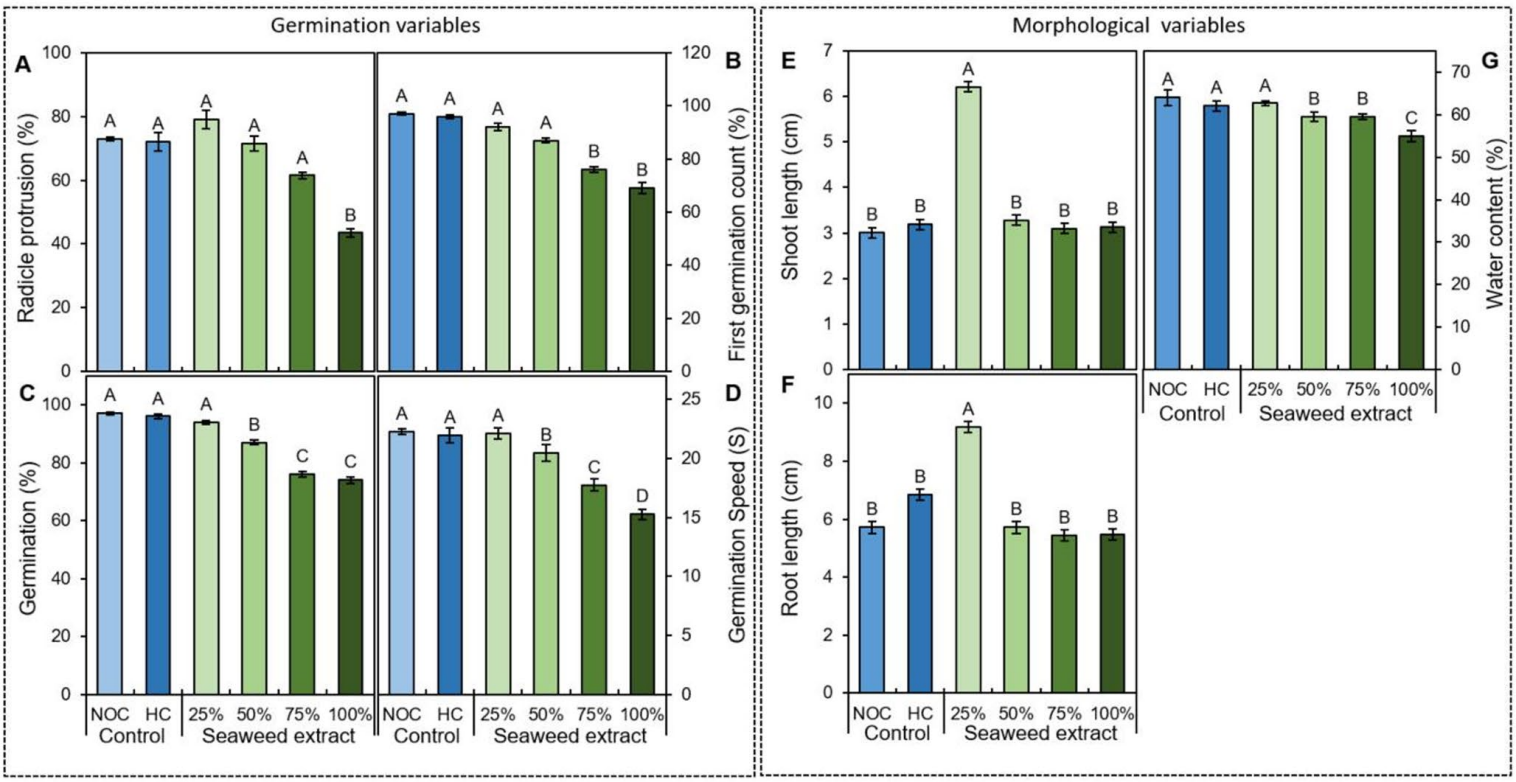
Germination (**A**–**D**) and morphological variables (**E**–**G**) of maize seeds and seedlings subjected to physiological conditioning with Kappaphycus alvarezii algal extract. **A** Radicle protrusion at 24 h; **B** first germination count at 4 days; **C** germination at 7 days; **D** germination speed index; **E** shoot length; **F** root length; **G** water content. NOC: No conditioning; HC: Hydroconditioning. Means followed by the same letter do not differ significantly according to the Scott-Knott test (*p* ≤ 0.05). Error bars represent standard error

Conditioning with both hydroconditioning and the marine extract increased the seed moisture content (from 8.97% in non-conditioned seeds to an average of 30.61% in conditioned seeds) compared to the non-conditioned control (Supplementary Table S4), whereas differences in final moisture content after drying were minimal among the conditioned treatments. After 48 h (Fig. 1A), radicle protrusion (*p* = 0.0005) was equivalent similar (mean of 71%) for non-conditioned seeds, hydroconditioning seeds and seeds treated with 25, 50 and 75% extract. In contrast, it was significantly reduced at the 100% concentration (44% exhibiting radicle protrusion). Initial germination count (*p* < 0.0001), performed on the fourth day (Fig. 1B), followed a similar pattern, with declines at 75% and 100%. Both final germination (10th day; *p* < 0.0001) (Fig. 1C) and the germination speed index (Fig. 1D; *p* < 0.0001) were highest in non-conditioned seeds, hydroconditioning seeds and 25% treatments. A decrease was observed starting at 50%, which became a sharp decline at 75% and 100%. Shoot length (Fig. 1E) and root length (Fig. 1F) were maximal at 25%, being greater than in all other treatments, which did not differ among themselves. Seedling water content (Fig. 1G) was higher in non-conditioned seeds, hydroconditioning seeds and 25% treatments and decreased above 25%; the 100% treatment showed a 39.72% reduction relative to non-conditioned seeds.

### Microcrustacean Acute Toxicity

**Fig. 2:**
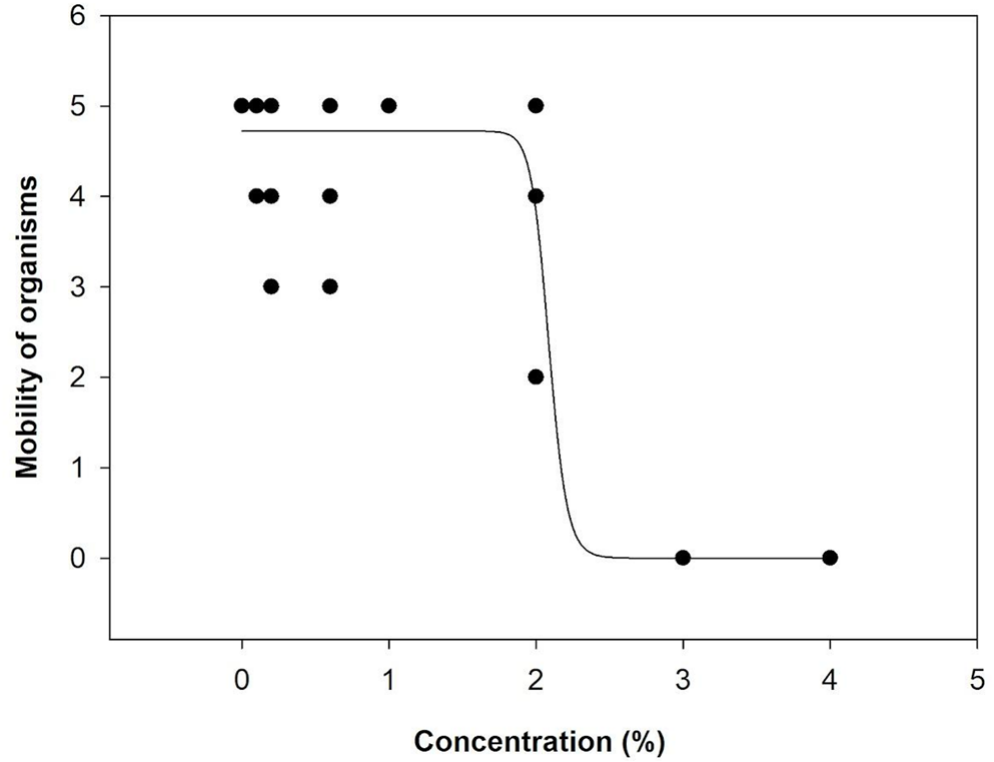
Mobility of *Ceriodaphnia silvestrii* organisms exposed to different concentrations of *Kappaphycus alvarezii* algal extract after 48 h exposure

Physicochemical parameters remained within acceptable ranges (pH 7.0–7.6; temperature 24.1–25 °C; hardness 40–44 mg CaCO3 L^−1^; dissolved O2 5.89–7.9 mg L^−1^) and control mortality was < 10%, in accordance with ABNT (2022). Conductivity increased with extract concentration (2390 µS cm^−1^ in the 4% solution). The reference NaCl test returned 48-h EC_50_ values between 1.42 and 1.68 g L^−1^, and the EC_50_ for the *Kappaphycus alvarezii* extract was 2.06% (EC_50_ 48 h = 2.06%; 95% CL, *p* = 0,0001) (Fig. 2). The statistical assumptions were verified, ensuring the validity of the comparisons made. The data met normality, variance tests and the EC_50_ was estimated with a 95% confidence interval.

## Discussion

The use of *K. alvarezii* extract at concentrations up to 50% did not enhance seed germination but showed performance equivalent to hydroconditioning and control treatments, with no statistical differences among them. Although no stimulatory effect was observed, the absence of inhibitory or phytotoxic responses at lower concentrations is noteworthy, particularly considering the use of genetically improved cultivars with high germination uniformity, which allows detection of subtle physiological variations induced by external agents (Bewley et al. 2013; Franzoni et al. 2023). However, concentrations ranging from 75–100% exhibited phytotoxic effects that impaired germination, likely due to phenolic compounds and polysaccharides (Vaghela et al. 2022).

The biphasic response pattern observed in Zea mays, with the 25% concentration promoting vegetative development, while higher concentrations (75–100%) inhibited germination, is consistent with the phenomenon of hormesis. This response is characterized by a stimulatory effect at low doses, where the extract acts as a biostimulant, while high doses provoke stress responses and induce phytotoxicity (Pannacci et al. 2022).

Seed conditioning with 25% extract promoted improvements in morphology and initial growth of both the shoot and root system, in line with studies attributing this effect to phytohormones and antioxidants that enhance nutrient uptake and increase tolerance to abiotic stress (Cocetta et al. 2022; Ahmad et al. 2022). The discrepancy between germination and vegetative development suggests that absorption and metabolism of bioactive compounds vary depending on phenological stage and administration route, involving hormones such as auxins and cytokinins (Arioli et al. 2015; Santner et al. 2009). At higher concentrations, antagonistic effects may occur, related to oxidative stress and excessive defense signaling, compromising photosynthesis, cell division, and growth. Consequently, germination and morphological parameters are affected, leading to detrimental effects on the plants, such as reduced or non-uniform germination, diminished root and shoot growth, and also a loss of productivity and vigor (Gill and Tuteja 2010; Petrov et al. 2015; Peleg and Blumwald 2011; Khan et al. 2009). Besides their effects on seed physiology, the environmental release of these extracts and leaching into aquatic environments warrants ecotoxicological considerations.

Ecotoxicological assessments showed that 2.06% of the extract caused toxicity in 50% of *C. silvestrii* individuals. This indicates that typical agronomic applications of 0.5–15% (Trivedi et al. 2023) could reach critical levels for sensitive aquatic organisms if leaching occurs. Toxicity may be associated with both the bioactive composition (phenolics, flavonoids, quinones, carotenoids, etc.) (Vaghela et al. 2022; Trivedi et al. 2023) and increased salinity, as conductivity reached 2390 µS cm^−1^ at 4% extract, a critical factor for cladocerans (Paturej and Gutkowska 2015). Water electrical conductivity is an indicator of water quality, and higher values can cause stress to aquatic organisms (Taniwaki et al. 2017). Santos et al. (2025) analyzed the effects of landscape structure in the Brazilian Cerrado on the diversity of aquatic communities and found that the richness and abundance of Cladocera decreased in streams with higher water conductivity. These results corroborate the tests performed, evidencing that the higher ionic/carrageenan load of the *Kappaphycus* extract may have a potential role as an osmotic stressor for the cladoceran *C. silvestrii*.

Toxicity tests with *Artemia salina* revealed that carrageenan, present in *K. alvarezii*, at 4.24 µg/mL induced 50% lethality (LC_50_) (Damayanti et al. 2025), demonstrating that even marine-adapted organisms can be significantly affected by concentration-dependent exposures. Moreover, the extract contains bioactive compounds with physiological activity (cytokinins, proteins, amino acids) and secondary metabolites with antimicrobial and antioxidant properties, such as phenolics, terpenes, and fatty acids (Trivedi et al. 2023). When released into water, these substances may act individually or synergistically, eliciting physiological and toxic responses in exposed organisms (Pérez et al. 2016). Furthermore, components from the extract have demonstrated general cytotoxic potential in other biological assays (Chang et al. 2017), suggesting that mechanisms of cell death may contribute to the observed toxicity.

Studies indicate that some plant extracts can be toxic to freshwater microcrustaceans. Santos et al. (2023) found that the leaf extract of *Annona squamosa* resulted in a 100% mortality rate for the microcrustacean Hyalella sp. Another study evaluated the toxic effect of the aqueous extract of Thuya (*Tetraclinis articulata*) on *Daphnia magna*, and acute toxicity and reprotoxicity were observed in the exposed organisms (Montassir et al. 2017). These studies indicate that bioextracts, although a more sustainable and less environmentally harmful agronomic alternative, can generate toxic effects in non-target populations.

Therefore, protocols for seed conditioning should include safe management of residual material, avoiding direct disposal into water bodies and promoting temporary storage or reuse to reduce environmental risks. While natural biostimulant use is regulated for agronomic efficacy, regulatory gaps remain regarding ecological impacts (Luo et al. 2021; Andrade et al. 2022), highlighting the need for policies that integrate evaluation of environmental behavior and aquatic ecosystem effects.

A discrepancy between beneficial agronomic doses and environmental safety is noted: the 25% extract solution, optimal for *Z. mays* growth, exhibited a concentration more than twelvefold higher than the 48-h EC_50_ (2.06%) for *C. silvestrii*. This renders the seed conditioning effluent unsuitable for disposal without prior treatment. This highlights an issue regarding “natural” biostimulants, which are not subjected to the rigorous ecotoxicological testing required for synthetic chemicals. Therefore, the standardization of aquatic ecotoxicity tests is necessary for biostimulant approval to establish clear protocols for managing agricultural effluents (Du Jardin 2015).

In conclusion, this study highlights that the application of *K. alvarezii* as a biostimulant must be guided by ecotoxicological metrics (such as EC_50_) to manage its inherent biphasic and toxic properties. Ultimately, defining a precise operational window, one that leverages agronomic benefits (hormesis) while strictly avoiding phytotoxicity and environmental hazards, is imperative for its viable use in sustainable agriculture.

## Conclusion

The results demonstrate organism-specific, dose-dependent effects. In *Zea mays*, a 25% concentration of *Kappaphycus alvarezii* extract acted as a biostimulant, enhancing germination and early seedling growth, whereas higher concentrations (≥ 50%) markedly reduced germination and seed water content, with no evident phytotoxic effects on seedling roots or shoots. In *Ceriodaphnia silvestrii*, exposure to the extract yielded an EC_50_ 2.06%, indicating acute risk to sensitive aquatic invertebrates. These findings underscore the need to integrate ecotoxicological assessments into approval and use protocols for marine-derived biostimulants, incorporating EC_50_ data into leaching exposure models and dosage guidelines to prevent contamination of water bodies. Mitigation strategies should include optimizing application volumes, establishing riparian buffer zones, and monitoring effluents, alongside targeted research to refine dosage protocols. Overall, the study provides an actionable basis to reconcile agronomic benefits with environmental safeguards and to inform more sustainable policy and practice for algal biostimulant use.

## Supplementary Information

Below is the link to the electronic supplementary material.

Supplementary file1 (PDF 530 kb)

## Data Availability

The datasets generated and/or analyzed during the current study are available from the corresponding author on reasonable request. Declarations.
