# Supplementary material for: Ecotoxicological Effect of Kappaphycus alvarezii Extract on Zea mays and Ceriodaphnia silvestrii
Source: Bull Environ Contam Toxicol. 2026 Mar 28;116(4):79. doi: 10.1007/s00128-026-04229-8 (PMC13032965; doi:10.1007/s00128-026-04229-8)
Supplement: Supplementary file 1 — Supplementary file1 (PDF 530 kb) [file 128_2026_4229_MOESM1_ESM.docx]

**Supplementary material**

**Ecotoxicological effect of *Kappaphycus alvarezii* extract on *Zea mays* and *Ceriodaphnia silvestrii***

Lucas Adriano Moreira^1^; Erika dos Santos Silva^1^; Antonio Rodrigues da Cunha Neto^1*^(0000-0001-7107-2755); Anelise Vieira Rosa Fernandes da Silva^1^; Alexandra dos Santos Ambrósio^1^; João Vitor Barbosa Calvelli^1^(0000-0001-6075-6158); Gabriela Ezequiel Costa Martins^1^; Maria José dos Santos-Wisniewski^1^; Breno Régis Santos^1^(0000-0002-3980-9013); Sandro Barbosa^1^(0000-0001-7321-0007)

^1^ Universidade Federal de Alfenas, Instituto de Ciências da Natureza, Alfenas-MG, Brazil.

* Corresponding author: antoniorodrigues.biologia@gmail.com

**Supplemental Table S1.** Typical composition (per liter) as listed in the product specifications of the commercial *Kappaphycus alvarezii* extract.

| **Nutrient** | | **Amino acid** | |
| --- | --- | --- | --- |
| Nitrogen (N) | 16.700 g | Glycine (Gly, G) | 14.4 g |
| Phosphorus (P) | 10.400 g | Proline (Pro, P) | 8.4 g |
| Boron (B) | 0.008 g | Alanine (Ala, A) | 5.6 g |
| Calcium (Ca) | 0.050 g | Glutamic acid (Glu, E) | 5.1 g |
| Magnesium (Mg) | 0.032 g | Aspartic acid (Asp, D) | 3.6 g |
| Sulfur (S) | 1.900 g | Arginine (Arg, R) | 4.2 g |
| Manganese (Mn) | 0.033 g | Serine (Ser, S) | 2.0 g |
| Zinc (Zn) | 0.006 g | Leucine (Leu, L) | 1.8 g |
| Copper (Cu) | 0.007 g | Lysine (Lys, K) | 2.3 g |
| Total Organic Carbon (TOC) | 41.500 g | Valine (Val, V) | 1.4 g |
| Potassium (K) | 4.900 g | Threonine (Thr, T) | 1.1 g |
| Iron (Fe) | 0.200 g | Phenylalanine (Phe, F) | 1.2 g |
|  |  | Isoleucine (Ile, I) | 0.9 g |
|  |  | Tyrosine (Tyr, Y) | 0.3 g |
|  |  | Histidine (His, H) | 0.4 g |

**Supplemental Table S2.** pH and electrical conductivity of the commercial *Kappaphycus alvarezii* extract and its tested dilutions for physiological conditioning of maize seeds.

| **Percentage of algal extract in solution** | **pH** | **Electrical conductivity (µS cm^-1^)** |
| --- | --- | --- |
| 100% | 5.53 | 62600 |
| 75% | 5.51 | 49200 |
| 50% | 5.53 | 37000 |
| 25% | 5.60 | 19160 |

**Supplemental Table S3.** Measured pH and electrical conductivity values of *Kappaphycus alvarezii* algal extract and its serial dilutions used in *Ceriodaphnia silvestrii* acute toxicity bioassays.

| **Percentage of algal extract in solution** | **pH** | **Electrical conductivity (µS cm^-1^)** |
| --- | --- | --- |
| 4% | 7.19 | 1919 |
| 3% | 7.25 | 1546 |
| 2% | 7.29 | 1253 |
| 1% | 7.36 | 1013 |
| 0.6% | 7.47 | 760 |
| 0.2% | 7.50 | 480 |
| 0.1% | 7.13 | 171 |

**Supplemental Table S4.** Moisture content of maize seeds subjected to physiological conditioning under different concentrations of *Kappaphycus alvarezii* seaweed extract, post-conditioning and post-drying.

| **Treatment** | **Moisture content post-conditioning (%)** | **Moisture content post-drying (%)** |
| --- | --- | --- |
| No conditioning | 8.97 | 10.50 |
| Hydroconditioning | 31.33 | 11.54 |
| 25% seaweed extract | 30.65 | 11.40 |
| 50% seaweed extract | 30.16 | 11.43 |
| 75% seaweed extract | 31.18 | 11.51 |
| 100% seaweed extract | 29.75 | 11.44 |
